# Diversification of East Asian subtropical evergreen broadleaved forests over the last 8 million years

**DOI:** 10.1002/ece3.9451

**Published:** 2022-10-30

**Authors:** Jun‐Wei Ye, De‐Zhu Li

**Affiliations:** ^1^ Germplasm Bank of Wild Species in Southwest China, Kunming Institute of Botany Chinese Academy of Sciences Kunming China; ^2^ Key Laboratory for Forest Resources Conservation and Utilization in the Southwest Mountains of China, Ministry of Education Southwest Forestry University Kunming China

**Keywords:** dominant species, fossil, late Miocene, Pleistocene, relict genera

## Abstract

The evolution of subtropical evergreen broadleaved forests (EBLFs) in East Asia is interesting while complicated. Genus‐level phylogenies indicate that the origins of EBLFs could trace back to the Oligocene–Miocene boundary or even the Eocene, while population‐level phylogeographic evidence suggests that they diversified after the Miocene, particularly in the Pleistocene. Here, we review the origins of dominant plant species to better understand the evolution of EBLFs. We compiled published estimates of the timing of origin of dominant species and diversification of evergreen relict genera from East Asian EBLFs. We also traced and visualized the evolution of EBLFs in the region using dated phylogenies and geographic distributions of the reviewed taxa. Most (76.1%) of the dominant species originated after the late Miocene, ca. 8 million years ago. Of the 10 evergreen relict genera, eight diverged near the late Miocene–Pliocene boundary or during the late Pliocene, and the remaining two diverged during the Pleistocene. Over the past 8 million years, geo‐climatic changes have triggered origins of most of the dominant EBLF species and provided refugia for evergreen relict genera. Three pulsed phases of evolution are suggested by genetic studies at the genus, species, and population levels. Fossil evidence and spatiotemporal investigations should be integrated to fully understand the evolution of EBLFs in East Asia.

## Graphical abstract

During the past 8 million years, geo‐climatic changes have triggered the origin of the most dominant EBLF species and have helped offer refugia for evergreen relict genera. Three different yet overlapping assembly stages can be identified at the generic, species, and population levels. Fossil and genetic investigations should be integrated to further explore the temporal and spatial dynamics of EBLF assembly during the late Cenozoic.

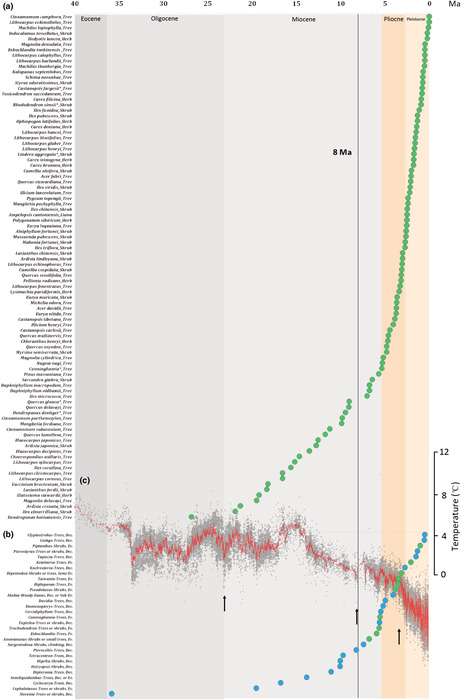

## 1 INTRODUCTION

Subtropical evergreen broadleaved forests (EBLFs) currently occur between 24–32°N latitude and 99–123°E longitude, and represent the most characteristic biome in East Asia (Wu, 1980; Song, 1988; Wu et al., 2010). These forests have high levels of species diversity and is one of the most important museum for Cenozoic relict species (Qiu et al., 2011). Its evolution is influenced by the East Asian monsoon, especially the summer monsoon (EASM) that brings a humid climate (Li, Luo, et al., 2021; Li, Valdes, et al., 2021; Wu, 1980; Wu et al., 2010). The evolution of EBLFs is a complicated and interesting issue in plant ecology and biogeography. Genetic studies at the genus or population levels have uncovered the evolution of EBLFs at different time scales.

Investigations at the genus level suggest that EBLFs originated near the Oligocene–Miocene boundary. Eocene origins of two dominant EBLF tree genera, *Cinnamomum* (Huang et al., 2016) and *Lithocarpus* (Yang et al., 2018), suggest an even earlier emergence of EBLFs. However, many more taxa originated in the late Oligocene or early Miocene, such as *Schima*, *Castanopsis* and *Machilus* (Yu et al., 2017), *Quercus* section *Cyclobalanopsis* (Deng et al., 2017), and *Dendrobium* (Xiang et al., 2016). The origin of the EBLFs is facilitated by evolution of the EASM (Deng et al., 2021; Yu et al., 2017). Although the EASM was suggested to have an Eocene origin (Licht et al., 2014), more convincing studies show that a monsoon pattern similar to that of the present in East Asia appeared between the Late Oligocene and the Early Miocene (Clift et al., 2015; Deng et al., 2021; Sun & Wang, 2005), Evolution of the angiosperm flora of China (Lu et al., 2018), or East Asian Flora (EAF; Chen et al., 2018) provide further insights into the origin of EBLFs. The dated phylogeny of the Chinese angiosperm genera show that 61% of the 1026 genera that occur only in eastern China (including northeastern, northern, and subtropical China) originated after 20 million years ago. Besides, age estimates of 213 clades (at the generic level or lower) for the EAF or 50 of the Sino‐Japanese forest subkingdom show that ca. 77% or 84% of the clades originated after 23 million years ago (Chen et al., 2018). Thus, these studies at generic/clades level also suggest that the Oligocene–Miocene boundary or earlier time period is of significance for the origin of EBLFs (Chen et al., 2018; Lu et al., 2018).

In contrast, population‐level phylogeographic studies suggest post‐Miocene divergence of EBLFs, particularly during the Pleistocene (Qiu et al., 2011; Ye et al., 2017). Some studies highlight the contribution of geo‐climate changes during the late Pliocene (ca. 3.6–2.6 million years ago) to genetic divergence (Huang et al., 2014; Kou et al., 2019; Wang et al., 2015), though the effects of Pleistocene climatic changes on EBLFs are likely quite complex (Qiu et al., 2011; Ye et al., 2017). Paleo‐biome reconstructions suggest that East Asian EBLFs retreated southward (<24°N) during glacial periods (Harrison et al., 2001; Ni et al., 2010), whereas phylogeographic studies suggest that plants survived in multiple northern (>24°N) refugia during glacial periods, with localized expansions during interglacial periods (Wang et al., 2015). Similar inter‐ and post‐glacial range expansions from southern refugia have been documented for some Lauraceae species (Fan et al., 2016; Ye et al., 2019), suggesting that glacial–interglacial cycles contributed to complex biographic and evolutionary dynamics during the Pleistocene (Fan et al., 2016).

The aim of this study was to improve EBLFs' evolution by reviewing published species‐level genetic investigations. Dominant species, for example, those with the highest abundance or highest biomass in a community, contribute significantly to the assembly and ecosystem function of EBLFs (Wang, 2006; Wu, 1980). Thus, we focused our review on studies of the origins of dominant species of three vegetation layers (tree, shrub, and herb). The radiations of relict genera (Tang et al., 2018) were also examined, because these taxa are highly significant in the evolutionary history of EBLFs even though they contribute minimally to the modern appearance of the vegetation (Qiu et al., 2017).

## 2 METHODS

### 2.1 The timing of origin and diversification

In the EBLFs, there are some 2900 flowering plant species, representing 147 families and 760 genera (Wang, 2006; Wu, 1980). Based on dominant species of different vegetation layers, there are 291 dominant species, which comprise ca. 1/10 of all EBLFs species (Wang, 2006). The 291 dominant species are composed of 152 trees, 89 shrubs, 29 herbs, and 21 lianas. According to Tang et al. (2018), there are 93 relict genera endemic to East Asia, and the subtropical region is the distribution center. The 93 genera are composed of 38 evergreen (including semi‐evergreen) and 55 deciduous genera.

The timing of origin of dominant species (mainly stem age with supplement of few crown age of intraspecies populations) and diversification of relict genera (crown age) was complied.

### 2.2 Illustration of EBLF evolution

To visually evaluate the evolution of EBLFs, we prepared a graphic showing characteristic dominant EBLF species from *Cinnamomum* and *Machilus* (Lauraceae), *Magnolia* and *Manglietia* (Magnoliaceae), *Quercus* and *Lithocarpus* (Fagaceae), and *Ilex* (Aquifoliaceae), as well as relict genera *Pseudotaxua* (Taxaceae), *Cunninghamia*, *Glyptostrobus* and *Taiwania* (Cupressaceae), *Trochodendron* (Trochodendraceae), and *Koelreuteria* (Sapindaceae). Time‐calibrated phylogenies of these genera were generated through Darwin Tree (http://www.darwintree.cn). Pictures and distribution information of dominant species and relict genera were obtained from the iPlant database (http://www.iplant.cn/). The distribution ranges were categorized into low (<25% EBLFs), medium (25%–50% EBLFs), and high (>50% EBLFs) levels. Stem/crown ages were calculated and are shown in Tables S1 and S2. All data were collected until September 1, 2020.

## 3 RESULTS

### 3.1 The timing of EBLF diversification

**FIGURE 1:**
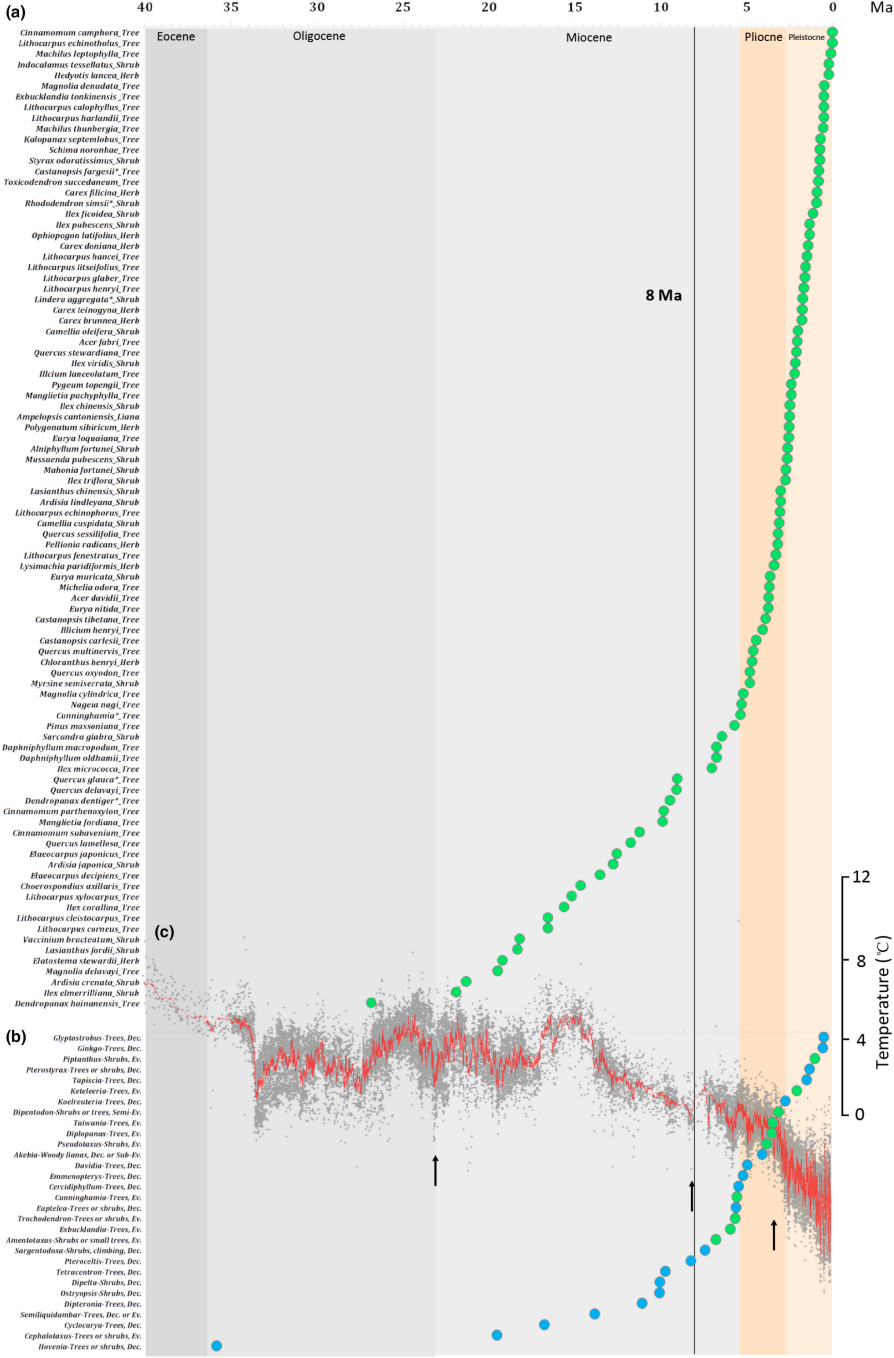
(a) Time of origin of 92 dominant species of East Asian subtropical evergreen broadleaved forests. The 95% highest probability‐density interval of origin time is not labeled as it was not often provided in the studies that provided the information. Asterisks indicate species for which the crown age is used. (b) Divergence times of 30 relict genera, with evergreen genera shown in green and deciduous genera in blue. (c) Stacked deep sea benthic foraminiferal oxygen‐isotope curve revealing dynamics of the global climate over the last 30 million years (Zachos et al., 2008). Black arrows indicate periods of intensification of the East Asian summer monsoon.

**FIGURE 2:**
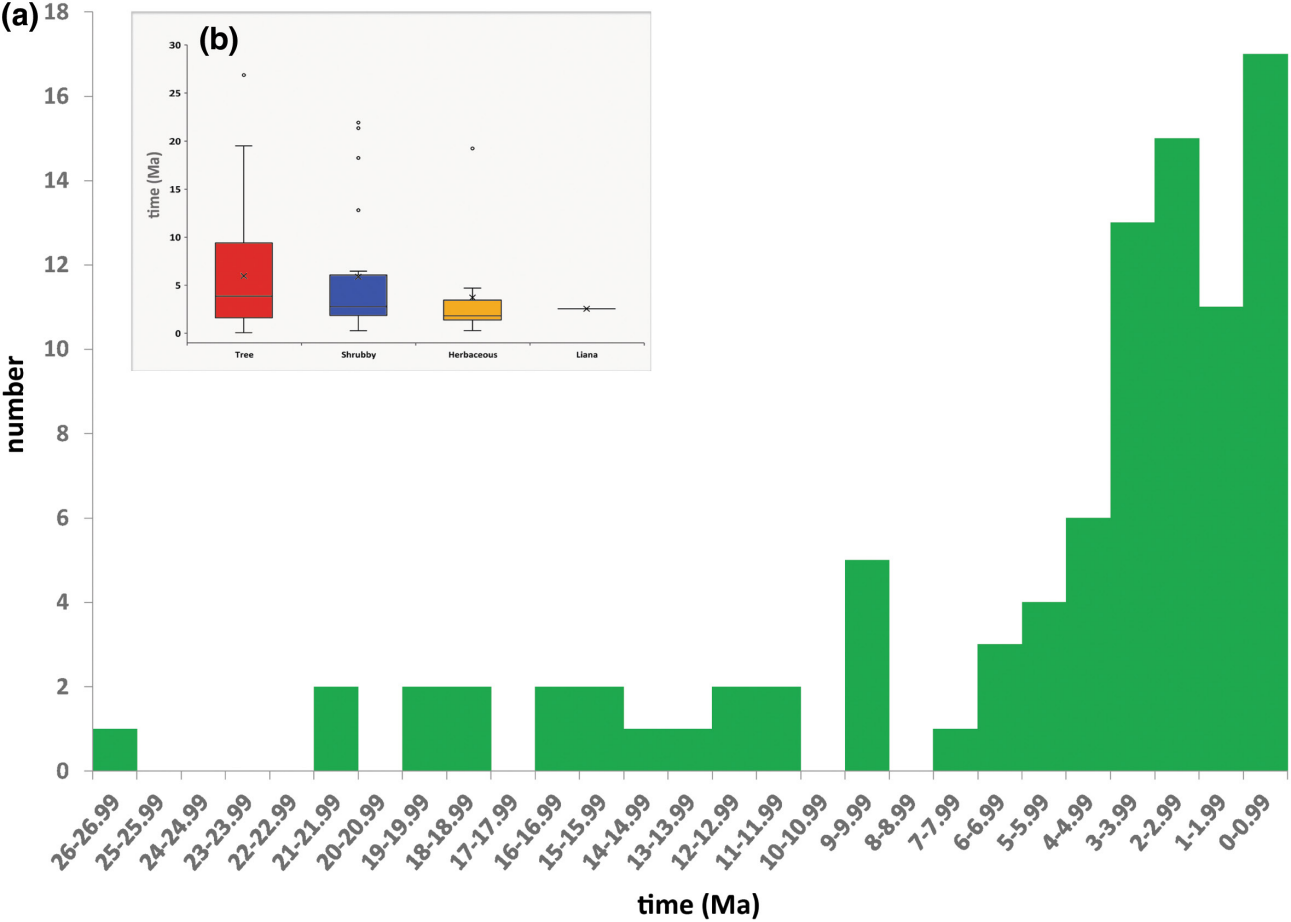
(a) Number of dominant species originating every million years and (b) comparison of origination times of the dominant tree, shrub, herbaceous, and liana species.

We compiled published estimates of the timing of origin for 92 of the 291 dominant species (Figure 1a), including 56 trees, 24 shrubs, 11 herbs, and 1 liana species. Of the 92 species for which data were available, 70 originated after the late Miocene, ca. 8 million years ago (Figure 1a, Table S1), with many species originating after 4 million years ago (Figures 1a and 2a). Both the median and average ages of origin were around 5 million years ago across all growth forms, although the tree species appear to be slightly older than shrub and herbaceous species (Figure 2b).

The crown ages of 30 sampled relict genera, which included 10 evergreen and 20 deciduous genera, ranged from the early Oligocene to the late Pleistocene (Table S2, Figure 1b). Of the 10 evergreen genera, four diverged around the late Miocene–Pliocene boundary, four diverged in the late Pliocene, and the remaining two originated in the Pleistocene (Figure 1b).

**FIGURE 3:**
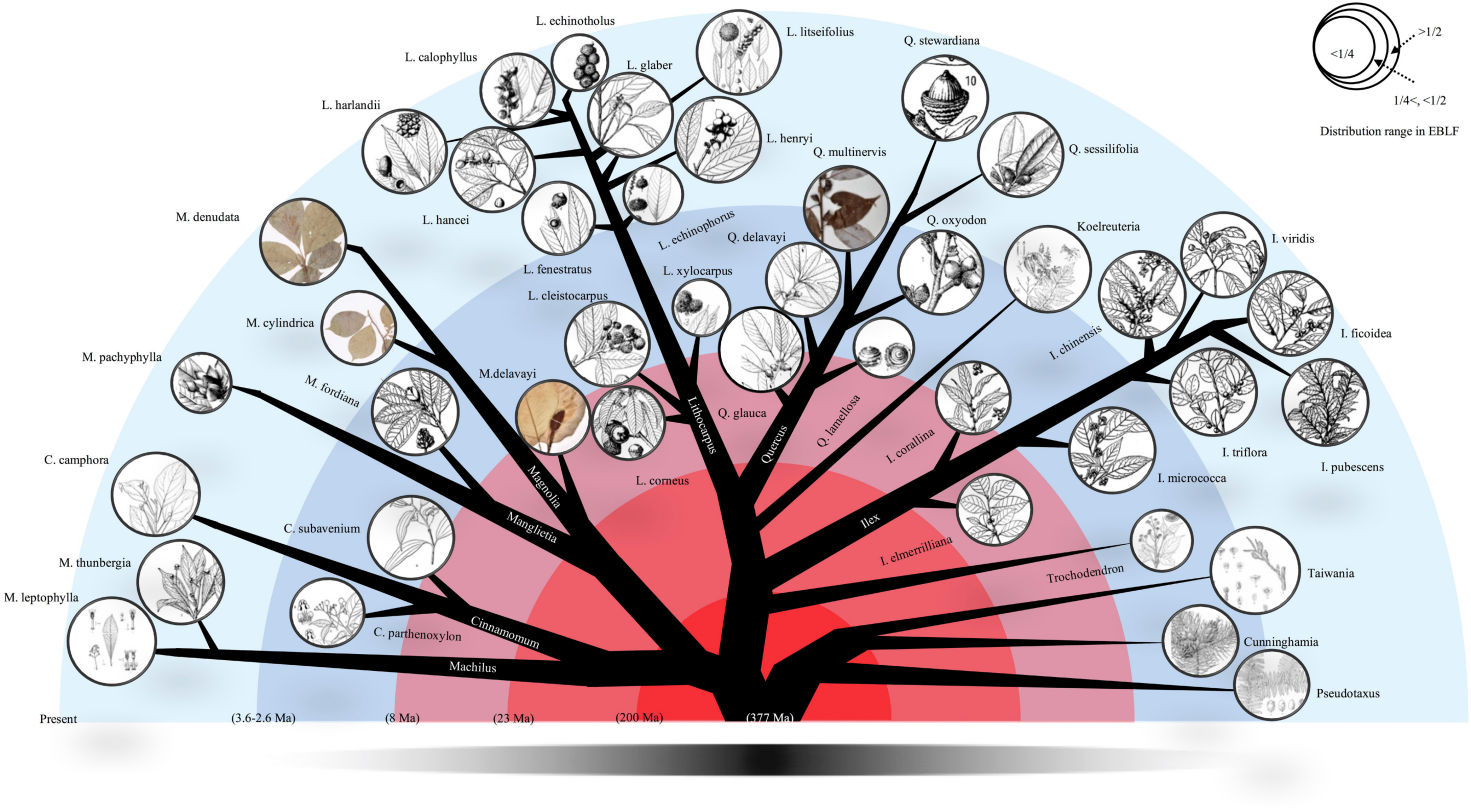
Illustration of the assembly of the East Asian subtropical evergreen broadleaved forests. Dated phylogenies of representative genera were generated with Darwin Tree (http://www.darwintree.cn). The stem/crown age of dominant species and relict genera are shown in Tables S1 and S2. Images and distribution information of different species were obtained from the iPlant database (http://www.iplant.cn/).

Based on the time‐calibrated phylogeny of select taxa (Figure S1), the last 8 million years was a critical time period for the diversification of EBLFs (Figure 3).

## 4 DISCUSSION

### 4.1 The last 8 million years were key to the diversification of EBLF species

We reviewed published species‐level data regarding the origins and diversification of key taxa to better understand the evolution of EBLFs, particularly because previous genus‐ and population‐level genetic studies suggest differing time estimates for the origins and diversification of these biomes (Deng et al., 2017; Qiu et al., 2011; Ye et al., 2017; Yu et al., 2017). Most (76.1%) of the sampled dominant species originated after the late Miocene (Figure 1a, Table S1) and the diversification of evergreen relict genera clustered around the late Miocene–Pliocene boundary and late Pliocene (Figure 1b, Table S2). Similar ages were inferred for all growth forms (Figure 2b), which is consistent with a suggestion by Lu et al. (2018) that herbaceous and woody genera accumulated at similar rates throughout geological time in eastern China, indicating that plants with different growth forms have likely evolved largely synchronously in EBLFs.

The recent origins (<8 million years ago) of so many dominant species in EBLFs highlight the importance of geo‐climate changes for speciation after the late Miocene (Figure 3). The evolution of EBLFs in East Asia has been strongly influenced by the East Asian monsoon, especially the EASM, which brings humid conditions (Li, Luo, et al., 2021; Li, Valdes, et al., 2021; Wu, 1980; Wu et al., 2010). Although it has been suggested that the EASM has Eocene origins (Licht et al., 2014), more convincing studies indicate that a monsoon pattern similar to the present pattern emerged between the late Oligocene and early Miocene (Clift et al., 2015; Deng et al., 2021; Sun & Wang, 2005). According to previous genus‐level biogeographic studies, diversification rates accelerated during the late Miocene, coincidentally with an intensified EASM in the late Miocene (An et al., 2001). Although possible changes to the EASM since the late Miocene are disputed (An et al., 2001; Chen et al., 2007), it probably intensified during the late Pliocene (An et al., 2001; Clift et al., 2015; Wan et al., 2006). Meanwhile, global cooling and the uplift of the Qinghai‐Tibet Plateau during the Pliocene may have further influenced genetic divergence and speciation rates (Huang et al., 2014; Kou et al., 2019; Wang et al., 2015). During the Pleistocene, population bottlenecks associated with inter‐ and post‐glacial northward expansions (Hewitt, 2004; Waters et al., 2013), or genetic isolation during high–low latitude shifts (Qiu et al., 2009, 2011), could also have provided abundant opportunities for speciation.

Divergence times and fossil distributions of the evergreen relict genera suggest that these plants found refugia and diversified after 8 million years ago (Figure 1b, Table S2). Fossil distributions indicate that the relict genera either migrated into EBLFs from high latitudes during or after the Miocene, or have been continuously present in this subtropical region since their origin (Manchester et al., 2009; Zhou & Momohara, 2005). For relict genera lacking fossil records in this region—such as *Amentotaxus*, *Diplopanax*, and *Taiwania*, whose youngest known fossils are from Miocene or late Pliocene sites of North America, Europe, or Japan (Manchester et al., 2009)—dated phylogenies suggest that they survived in EBLF refugia after the Miocene–Pliocene boundary (*Amentotaxus*) or late Pliocene (*Diplopanax* and *Taiwania*; Table S2). For relict genera with rich fossil records from subtropical regions—such as *Cunninghamia*, recently documented from the late Miocene in southeastern China (Du et al., 2012)—genetic imprints generally only trace back to the Miocene–Pliocene boundary (Table S2). With an increasingly humid climate after the late Miocene (An et al., 2001; Clift et al., 2015; Wan et al., 2006) and the absence of large ice sheets in the Pleistocene (Qiu et al., 2017), evergreen relict genera were likely able to thrive in EBLFs. Meanwhile, global cooling (Huang et al., 2015) and Pleistocene glaciation (Hewitt, 2004) resulted in extensive contractions and extinctions of evergreen species outside of EBLFs, especially in Europe (Kovar‐Eder et al., 2006; Svenning, 2003).

Attention should be paid to the discordance of time between fossil emergence and dated origin. Fossils can be well behind the dated origin, such as Qian (2019) found 77% genera (ca. 140) that are dated younger than the Miocene of Lu et al. (2018) has fossils before the Miocene. Older origin than fossils may due to sparsity or less sampling of fossils (Li et al., 2019; Manchester et al., 2009). We also found that the diversifications of relict genera show lags behind the first appearance of fossils.

### 4.2 EBLF evolution is dynamic and time dependent

The evolution of modern floras can be reconstructed using dated phylogenies (Lu et al., 2018), while the evolution of ancient floras is better investigated using fossils (Manchester et al., 2009; Zhou & Momohara, 2005). In many ways, EBLFs are complex mixtures of modern and ancient floras (Figure 3) as they harbor many relicts, notably *Ginkgo*, *Metasequoia*, *Glyptostrobus*, *Taiwania* or *Liriodendron*, *Davidia*, and others (Ying & Chen, 2011), all of which have very old stem ages, yet the extant populations of the relict Ginkgo have diverged as recently as 0.39 million years ago (Hohmann et al., 2018). Thus, EBLFs have served as both museums and cradles (Lu et al., 2018).

The evolution of EBLFs can be further investigated using fossil and palynological evidence. Some gymnosperm species have fossil records that date back to the Cretaceous, indicating widespread historical distributions (*Cunninghamia* and *Taiwania*; Chou et al., 2011; Manchester et al., 2009). Among angiosperms, late Cretaceous fossils from China include *Dianthus*, *Liquidambar*, and *Altingia* (Ying & Chen, 2011). Integrated modeling of climate, vegetation, and plant diversity data with fossil evidence suggests that the uplift of northern Tibet during the Paleogene and Neogene spurred a transition from deciduous broadleaf vegetation to evergreen broadleaf vegetation, with increases in plant diversity across southeastern Asia (Li, Luo, et al., 2021; Li, Valdes, et al., 2021). For example, fossils indicate the presence of subtropical EBLFs in Southwest China (the Jianchuan basin) during the Eocene (Gourbet et al., 2017), and paleovegetation reconstruction (Sun et al., 2011) and palynological data (Zhang et al., 2012) suggest that subtropical EBLFs existed in Southwest China during the Miocene as well. After the Pliocene, fossil assemblages with compositions similar to modern EBLFs are well documented (Wu, 1980; Wu et al., 2010), indicating recent diversification of the modern biome.

The evolution of the modern EBLF biome likely occurred in three pulsed phases. Previous genus‐level dated phylogenies traced the early stages of EBLF evolution to the Eocene and Oligocene–Miocene boundary, with accelerated diversification during the late Miocene (Deng et al., 2017; Xiang et al., 2016; Yu et al., 2017). In contrast, population‐level phylogeographic studies document more recent EBLF diversification after the Miocene and especially in the Pleistocene (Qiu et al., 2011; Ye et al., 2017). The species‐level review presented here focuses on reconstructing EBLF diversification during the intermediate period (Figures 1 and 2). A large portion of dominant species (39/92, 42%) originated during the Pleistocene, with a minority of species (22/92, 23.9%) originating before the late Miocene, with the earliest origin in the Oligocene (Figure 1). A small proportion of genera also originated after the late Miocene in eastern China (~36%; Lu et al., 2018) or the Sino‐Japanese forest subkingdom (~38%; Chen et al., 2018). According to phylogeographic studies, geo‐climatic changes in the Miocene and Pliocene triggered the diversification of plants in broadleaved forests in subtropical China (Ye et al., 2017). However, the distribution of origins before the late Miocene is more discrete than that after late Miocene (Figures 1a and 2a), which may suggest that the early stage of EBLFs evolution was more dynamic and only some portion of ancestral taxa have successfully survived (López‐Pujol et al., 2011).

## 5 CAVEATS

A caveat regarding our findings is that our sampling is limited. For example, only ~30% of dominant evergreen species (representing just ~3% of all evergreen species) were used to infer timing of diversification of EBLF species. However, we suggest that the majority of EBLF species also originated after the late Miocene, as diversification rates accelerated in different clades during the late Miocene (Deng et al., 2017; Wang et al., 2020; Yu et al., 2017). Similarly, we suspect that increased sampling would not change the predicted time of diversification for evergreen relict genera, as estimates of the earliest diversification of deciduous relict genera trace back to the early Oligocene (Figure 1).

The second caveat concerns the time is compiled through different studies using different markers and fossils/calibrations. In the future, a dated phylogenetic tree of all subtropical evergreen species should be established using increased barcoding (Liu et al., 2015) or plastome sequencing (Jin et al., 2021). Because modern barcoding methods have different resolution in different genera, plastome sequencing is recommended as a superior method for constructing such a super‐tree (Li, Luo, et al., 2021; Li, Valdes, et al., 2021).

Lastly, attention should be paid to the inconsistencies between fossil and genetic evidence. Several fossil sited and many evergreen elements have been found in EBLFs, such as *Castanopsis nanningensis*, *C. guangxiensis*, *Elaeocarpus nanningensis*, and *Lithocarpoxylon nanningensis* in the Yongning Formation (Huang et al., 2018); *Mahonia ningmingensis* in the Ningming Formation (Hu et al., 2017); *Elaeocarpus presikkimensis* in the Erzitang Formation; and *Elaeocarpus prerugosus*, *E. prelacunosus*, *E. preserratus*, and *E. preprunifolioides* in the Foluo Formation (Liu et al., 2022). Geological ages of these formations range from the Oligocene to the late Miocene (Hu et al., 2017; Huang et al., 2018; Liu et al., 2022), indicating that EBLFs may have even earlier origins than suggested by phylogenetic reconstructions based on genetic data.

## 6 CONCLUSION

Climatic changes in the Eocene or Oligocene–Miocene boundary played an important role in the evolution of East Asian EBLFs. The evolution of modern EBLFs occurred in three pulsed phases, as revealed by studies at the genus, species, and population levels. Integrating multiple lines of evidence, we suggest that the last 8 million years were highly significant in the diversification of EBLFs.

## AUTHOR CONTRIBUTIONS

**Jun‐Wei Ye:** Conceptualization (equal); funding acquisition (equal); methodology (equal); project administration (equal); writing – original draft (equal); writing – review and editing (equal). **De‐Zhu Li:** Conceptualization (equal); formal analysis (equal); methodology (equal); project administration (equal); writing – original draft (equal); writing – review and editing (equal).

## Supporting information


Table S1
Table S2
Figure S1

## Data Availability

Origin and diversification times were compiled from references in Tables S1 and S2. The time‐calibrated phylogeny was generated through Darwin Tree (http://www.darwintree.cn). Pictures and distribution information for all taxa were obtained from the iPlant database (http://www.iplant.cn/).
