# Supplementary material for: Diversification of East Asian subtropical evergreen broadleaved forests over the last 8 million years
Source: Ecol Evol. 2022 Oct 30;12(11):e9451. doi: 10.1002/ece3.9451 (PMC9618824; doi:10.1002/ece3.9451)
Supplement: Supplementary file 1 — Table S1 Table S2 Figure S1 [file ECE3-12-e9451-s001.docx]

**Supporting Information**

**Diversification of East Asian subtropical evergreen broadleaved forests over the last eight million years**

Jun-Wei Ye and De-Zhu Li

| **Table S1** The timing of 92 dominant species in East Asian subtropical evergreen broadleaved forests. | | | | | | | |
| --- | --- | --- | --- | --- | --- | --- | --- |
| Species | Family | Life form | Stem age (Ma) | 95% HPD (Ma) | Marker | Divergence time calibrations | Reference |
| *Cinnamomum camphora* | Lauraceae | Tree | 0.06 | (0-0.25) | ITS, *RPB2, LEAFY* | One fossil and one geographic event | ([Huang *et al.*, 2016](#_ENREF_7)) |
| *Lithocarpus echinotholus* | Fagaceae | Tree | 0.06 | ns | ITS | One secondary calibration point | ([Yang *et al.*, 2018](#_ENREF_38)) |
| *Machilus leptophylla* | Lauraceae | Tree | 0.14 | ns | *atpB, matK, ndhF, rbcL, matR* | 138 fossils | ([Lu *et al.*, 2018](#_ENREF_18)) |
| *Indocalamus tessellatus* | Gramineae | Shrub | 0.26 | (0-1.09) | *rbcL-psaI, rpl32-trnL, rps15-ndhF, trnG-trnT, trnT-trnL, ycf4-cemA* | Four fossils | ([Zhang, XZ *et al.*, 2016](#_ENREF_47)) |
| *Hedyotis lancea* | Rubiaceae | Herb | 0.27 | (0-1.26) | *rps16, atpB-rbcL, trnL-F, petD* | Six fossils | ([Janssens *et al.*, 2016](#_ENREF_9)) |
| *Magnolia denudata* | Magnoliaceae | Tree | 0.51 | ns | *PHYA, LFY, GAI* | Three fossils | ([Nie *et al.*, 2008](#_ENREF_23)) |
| *Exbucklandia tonkinensis* | Hamamelidaceae | Tree | 0.54 | ns | *trnL-F, psaA-ycf3, rps16, matK, atpB-rbcL, psbA-trnH* | Four fossils | ([Xie *et al.*, 2010](#_ENREF_36)) |
| *Lithocarpus calophyllus* | Fagaceae | Tree | 0.55 | ns | ITS | One secondary calibration point | ([Yang *et al.*, 2018](#_ENREF_38)) |
| *Lithocarpus harlandii* | Fagaceae | Tree | 0.55 | ns | ITS | One secondary calibration point | ([Yang *et al.*, 2018](#_ENREF_38)) |
| *Machilus thunbergia* | Lauraceae | Tree | 0.59 | ns | *atpB, matK, ndhF, rbcL, matR* | 138 fossils | ([Lu *et al.*, 2018](#_ENREF_18)) |
| *Kalopanax septemlobus** | Araliaceae | Tree | 0.74 | (0.11-1.53) | *trnH-trnA, trnS-trnfM, rpl32-trnL* | Substitution rate | ([Sakaguchi *et al.*, 2012](#_ENREF_25)) |
| *Schima noronhae* | Theaceae | Tree | 0.77 | ns | Plastid genome | Eight fossils | ([Yu *et al.*, 2017](#_ENREF_42)) |
| *Styrax odoratissimus* | Styracaceae | Shrub | 0.78 | ns | *atpB, matK, ndhF, rbcL, matR* | 138 fossils | ([Lu *et al.*, 2018](#_ENREF_18)) |
| *Castanopsis fargesii** | Fagaceae | Tree | 0.83 | (0.16-1.9) | Chloroplast microsatellites | Substitution rate | ([Sun, Y *et al.*, 2014](#_ENREF_28)) |
| *Toxicodendron* *succedaneum* | Anacardiaceae | Tree | 0.86 | ns | ETS, *trnL-F, rps16* | Three fossils | ([Weeks *et al.*, 2014](#_ENREF_33)) |
| *Carex filicina* | Cyperaceae | Herb | 0.94 | ns | *matK*, ITS, ETS | Ten fossils | ([Martín-Bravo *et al.*, 2019](#_ENREF_20)) |
| *Rhododendron simsii** | Ericaceae | Shrub | 0.97 | (0.94-0.97) | *rpl20-rps12, trnL-F* | Substitution rate | ([Li *et al.*, 2012](#_ENREF_15)) |
| *Ilex ficoidea* | Aquifoliaceae | Shrub | 1.18 | (0.18-3.17) | ITS, *nepGS* | Three fossils | ([Yao *et al.*, 2020](#_ENREF_39)) |
| *Ilex pubescens* | Aquifoliaceae | Shrub | 1.37 | (0.01-7.15) | ITS, *nepGS* | Three fossils | ([Yao *et al.*, 2020](#_ENREF_39)) |
| *Ophiopogon latifolius* | Liliaceae | Herb | 1.38 | (0.41-2.62) | ITS | One secondary calibration point and two fossil | ([Wang & Yang, 2018](#_ENREF_31)) |
| *Carex doniana* | Cyperaceae | Herb | 1.45 | ns | *matK,* ITS, ETS | Ten fossils | ([Martín-Bravo *et al.*, 2019](#_ENREF_20)) |
| *Lithocarpus hancei* | Fagaceae | Tree | 1.53 | ns | ITS | One secondary calibration point | ([Yang *et al.*, 2018](#_ENREF_38)) |
| *Lithocarpus litseifolius* | Fagaceae | Tree | 1.6 | ns | ITS | One secondary calibration point | ([Yang *et al.*, 2018](#_ENREF_38)) |
| *Lithocarpus glaber* | Fagaceae | Tree | 1.65 | ns | ITS | One secondary calibration point | ([Yang *et al.*, 2018](#_ENREF_38)) |
| *Lithocarpus henryi* | Fagaceae | Tree | 1.71 | ns | ITS | One secondary calibration point | ([Yang *et al.*, 2018](#_ENREF_38)) |
| *Lindera aggregata* | Lauraceae | Shrub | 1.78 | 0.67-3.36 | *rpl16, psbA–trnH, trnL–trnF, trnS–trnG* | Three fossils and one geological event | ([Ye *et al.*, 2019](#_ENREF_40)) |
| *Carex teinogyna* | Cyperaceae | Herb | 1.8 | ns | *matK,* ITS, ETS | Ten fossils | ([Martín-Bravo *et al.*, 2019](#_ENREF_20)) |
| *Carex brunnea* | Cyperaceae | Herb | 1.82 | ns | *matK,* ITS, ETS | Ten fossils | ([Martín-Bravo *et al.*, 2019](#_ENREF_20)) |
| *Camellia oleifera* | Theaceae | Shrub | 2.06 | ns | Plastid genome | Eight fossils | ([Yu *et al.*, 2017](#_ENREF_42)) |
| *Acer fabri* | Sapindaceae | Tree | 2.1 | ns | *atpB, matK, ndhF, rbcL, matR* | 138 fossils | ([Lu *et al.*, 2018](#_ENREF_18)) |
| *Quercus stewardiana* | Fagaceae | Tree | 2.14 | (0.07-3.21) | RAD-seq | Two fossils | ([Deng *et al.*, 2017](#_ENREF_5)) |
| *Ilex viridis* | Aquifoliaceae | Shrub | 2.2 | (0.02-6.05) | ITS, *nepGS* | Three fossils | ([Yao *et al.*, 2020](#_ENREF_39)) |
| *Illcium lanceolatum* | Magnoliaceae | Tree | 2.24 | ns | *trnL-F, trnG,* ITS | Two fossils | ([Morris *et al.*, 2007](#_ENREF_21)) |
| *Pygeum topengii* | Rosaceae | Tree | 2.43 | ns | *rps16, rpl16,* ITS | One secondary calibration point and one fossil | ([Liu *et al.*, 2013](#_ENREF_17)) |
| *Manglietia pachyphylla* | Magnoliaceae | Tree | 2.44 | ns | *PHYA, LFY, GAI* | Three fossils | ([Nie *et al.*, 2008](#_ENREF_23)) |
| *Ilex chinensis* | Aquifoliaceae | Shrub | 2.53 | (1.13-4.61) | ITS, *nepGS* | Three fossils | ([Yao *et al.*, 2020](#_ENREF_39)) |
| *Ampelopsis cantoniensis* | Vitaceae | Liana | 2.55 | (0.41-5.55) | *rps16, trnL-F, atpB-rbcL, psbA-trnH* | One secondary calibration point and one fossil | ([Nie *et al.*, 2012](#_ENREF_22)) |
| *Polygonatum sibiricum* | Liliaceae | Herb | 2.56 | ns | ITS, *rpl16, trnL-F, psbA-trnH* | Two secondary calibration points | ([Kim *et al.*, 2017](#_ENREF_10)) |
| *Eurya loquaiana* | Pentaphylacaceae | Tree | 2.58 | ns | *trnL-F, aptB-rbcL,* ITS | One fossil | ([Su *et al.*, 2011](#_ENREF_27)) |
| *Alniphyllum fortunei* | Styracaceae | Shrub | 2.66 | ns | *atpB, matK, ndhF, rbcL, matR* | 138 fossils | ([Lu *et al.*, 2018](#_ENREF_18)) |
| *Mussaenda pubescens* | Rubiaceae | Shrub | 2.68 | (0.55-6.58) | *rps16, atpB-rbcL, trnL-F, petD* | Six fossils | ([Janssens *et al.*, 2016](#_ENREF_9)) |
| *Mahonia fortunei* | Berberidaceae | Shrub | 2.76 | (0.05-4.65) | ITS, *accD, ndhF, psbA-trnH, rbcL* | One secondary calibration point and one fossil | ([Yu & Chung, 2017](#_ENREF_41)) |
| *Ilex triflora* | Aquifoliaceae | Shrub | 2.77 | (0.76-6.05) | ITS, *nepGS* | Three fossils | ([Yao *et al.*, 2020](#_ENREF_39)) |
| *Lasianthus chinensis* | Rubiaceae | Shrub | 3.05 | ns | *rps16, trnL-F,* ITS, ETS | One secondary calibration point and one fossil | ([Smedmark *et al.*, 2014](#_ENREF_26)) |
| *Ardisia lindleyana* | Primulaceae | Shrub | 3.06 | ns | *atpB, matK, ndhF, rbcL, matR* | 138 fossils | ([Lu *et al.*, 2018](#_ENREF_18)) |
| *Lithocarpus echinophorus* | Fagaceae | Tree | 3.1 | ns | ITS | One secondary calibration point | ([Yang *et al.*, 2018](#_ENREF_38)) |
| *Camellia cuspidata* | Theaceae | Shrub | 3.15 | ns | Plastid genome | Eight fossils | ([Yu *et al.*, 2017](#_ENREF_42)) |
| *Quercus sessilifolia* | Fagaceae | Tree | 3.21 | (0.01-4.74) | RAD-seq | Two fossils | ([Deng *et al.*, 2017](#_ENREF_5)) |
| *Pellionia radicans* | Urticaceae | Herb | 3.23 | ns | *trnL-trnF, rpl14-rps8-infA-rpl36, matK, rbcL, I*TS, 18S, *matR* | Five fossils | ([Wu *et al.*, 2018](#_ENREF_35)) |
| *Lithocarpus fenestratus* | Fagaceae | Tree | 3.33 | ns | ITS | One secondary calibration point | ([Yang *et al.*, 2018](#_ENREF_38)) |
| *Lysimachia paridiformis* | Primulaceae | Herb | 3.45 | (1.79-4.92) | ITS, *matK, rbcL, trnL-F, trnH-psbA, rpl20-rps12, atpF-atpH, atpB-rbcL, rps16, trnS-trnG, rpl32-trnL* | Two secondary calibration points | Yan et al., 2018 |
| *Eurya muricata* | Pentaphylacaceae | Shrub | 3.67 | ns | *trnL-F, aptB-rbcL,* ITS | One fossil | ([Su *et al.*, 2011](#_ENREF_27)) |
| *Michelia odora* | Magnoliaceae | Tree | 3.71 | ns | *PHYA, LFY, GAI* | Three fossils | ([Nie *et al.*, 2008](#_ENREF_23)) |
| *Acer davidii* | Sapindaceae | Tree | 3.75 | ns | *atpB, matK, ndhF, rbcL, matR* | 138 fossils | ([Lu *et al.*, 2018](#_ENREF_18)) |
| *Eurya nitida* | Pentaphylacaceae | Tree | 3.78 | ns | *trnL-F, aptB-rbcL,* ITS | One fossil | ([Su *et al.*, 2011](#_ENREF_27)) |
| *Castanopsis tibetana* | Fagaceae | Tree | 3.94 | ns | *atpB, matK, ndhF, rbcL, matR* | 138 fossils | ([Lu *et al.*, 2018](#_ENREF_18)) |
| *Illicium henryi* | Magnoliaceae | Tree | 4.11 | ns | *trnL-F, trnG,* ITS | Two fossils | ([Morris *et al.*, 2007](#_ENREF_21)) |
| *Castanopsis carlesii* | Fagaceae | Tree | 4.49 | ns | *atpB, matK, ndhF, rbcL, matR* | 138 fossils | ([Lu *et al.*, 2018](#_ENREF_18)) |
| *Quercus multinervis* | Fagaceae | Tree | 4.65 | (0-6.14) | RAD-seq | Two fossils | ([Deng *et al.*, 2017](#_ENREF_5)) |
| *Chloranthus henryi* | Chloranthaceae | Herb | 4.71 | ns | rbcL, trnL-F, rpl20-rps12, rps16 | One secondary calibration point and two fossil | ([Zhang, Q *et al.*, 2015](#_ENREF_46)) |
| *Quercus oxyodon* | Fagaceae | Tree | 4.84 | (0.01-6.19) | RAD-seq | Two fossils | ([Deng *et al.*, 2017](#_ENREF_5)) |
| *Myrsine semiserrata* | Primulaceae | Shrub | 4.85 | (2.14-7.02) | ITS, *matK, rbcL, trnL-F, trnH-psbA, rpl20-rps12, atpF-atpH, atpB-rbcL, rps16, trnS-trnG, rpl32-trnL* | Two secondary calibration points | Yan et al., 2018 |
| *Magnolia cylindrica* | Magnoliaceae | Tree | 5.23 | ns | *PHYA, LFY, GAI* | Three fossils | ([Nie *et al.*, 2008](#_ENREF_23)) |
| *Nageia nagi* | Podocarpaceae | Tree | 5.33 | ns | *rbcL, matK,* 18S, *PHYP* | Sixteen fossils | ([Leslie *et al.*, 2012](#_ENREF_13)) |
| *Cunninghamia** | Cupressaceae | Tree | 5.41 | ns | *rbcL, matK,* 18S, *PHYP* | Sixteen fossils | ([Leslie *et al.*, 2012](#_ENREF_13)) |
| *Pinus massoniana* | Pinaceae | Tree | 5.74 | ns | *rbcL, matK,* 18S, *PHYP* | Sixteen fossils | ([Leslie *et al.*, 2012](#_ENREF_13)) |
| *Sarcandra glabra* | Chloranthaceae | Shrub | 6.47 | ns | *rbcL, trnL-F, rpl20-rps12, rps16* | One secondary calibration point and two fossil | ([Zhang, Q *et al.*, 2015](#_ENREF_46)) |
| *Daphniphyllum macropodum* | Daphniphyllaceae | Tree | 6.79 | ns | *atpB, matK, ndhF, rbcL, matR* | 138 fossils | ([Lu *et al.*, 2018](#_ENREF_18)) |
| *Daphniphyllum oldhamii* | Daphniphyllaceae | Tree | 6.79 | ns | *atpB, matK, ndhF, rbcL, matR* | 138 fossils | ([Lu *et al.*, 2018](#_ENREF_18)) |
| *Ilex micrococca* | Aquifoliaceae | Tree | 7.07 | (4.47-12.04) | ITS, *nepGS* | Three fossils | ([Yao *et al.*, 2020](#_ENREF_39)) |
| *Quercus glauca** | Fagaceae | Tree | 9.07 | (5.16-13.32) | *trnH-psbA, trnT-trnL, atpI-atpH* | Four fossils | ([Xu *et al.*, 2015](#_ENREF_37)) |
| *Quercus delavayi* | Fagaceae | Tree | 9.12 | (0.12-11.63) | RAD-seq | Two fossils | ([Deng *et al.*, 2017](#_ENREF_5)) |
| *Dendropanax dentiger** | Araliaceae | Tree | 9.49 | ns | *ndhF, trnL-F, rps16, atpB-rbcL, rpl16, psbA-trnH,* ITS | Two fossils | ([Li & Wen, 2013](#_ENREF_14)) |
| *Cinnamomum parthenoxylon* | Lauraceae | Tree | 9.86 | (3.81-17.32) | ITS, *RPB2, LEAFY* | One fossil and one geographic event | ([Huang *et al.*, 2016](#_ENREF_7)) |
| *Manglietia fordiana* | Magnoliaceae | Tree | 9.93 | ns | *PHYA, LFY, GAI* | Three fossils | ([Nie *et al.*, 2008](#_ENREF_23)) |
| *Cinnamomum subavenium* | Lauraceae | Tree | 11.27 | (4.93-19.44) | ITS, *RPB2, LEAFY* | One fossil and one geographic event | ([Huang *et al.*, 2016](#_ENREF_7)) |
| *Quercus lamellosa* | Fagaceae | Tree | 11.77 | (9.72-14.23) | RAD-seq | Two fossils | ([Deng *et al.*, 2017](#_ENREF_5)) |
| *Elaeocarpus japonicus* | Elaeocarpaceae | Tree | 12.59 | ns | *atpB, matK, ndhF, rbcL and matR* | 138 fossils | ([Lu *et al.*, 2018](#_ENREF_18)) |
| *Ardisia japonica* | Primulaceae | Shrub | 12.81 | ns | *atpB, matK, ndhF, rbcL and matR* | 138 fossils | ([Lu *et al.*, 2018](#_ENREF_18)) |
| *Elaeocarpus decipiens* | Elaeocarpaceae | Tree | 13.57 | ns | *atpB, matK, ndhF, rbcL and matR* | 138 fossils | ([Lu *et al.*, 2018](#_ENREF_18)) |
| *Choerospondias axillaris* | Anacardiaceae | Tree | 14.7 | ns | ETS, *trnL-F, rps16* | Three fossils | ([Weeks *et al.*, 2014](#_ENREF_33)) |
| *Lithocarpus xylocarpus* | Fagaceae | Tree | 15.21 | ns | ITS | One secondary calibration point | ([Yang *et al.*, 2018](#_ENREF_38)) |
| *Ilex corallina* | Aquifoliaceae | Tree | 15.65 | (9.57-24.63) | ITS, *nepGS* | Three fossils | ([Yao *et al.*, 2020](#_ENREF_39)) |
| *Lithocarpus cleistocarpus* | Fagaceae | Tree | 16.59 | ns | ITS | One secondary calibration point | ([Yang *et al.*, 2018](#_ENREF_38)) |
| *Lithocarpus corneus* | Fagaceae | Tree | 16.59 | ns | ITS | One secondary calibration point | ([Yang *et al.*, 2018](#_ENREF_38)) |
| *Vaccinium bracteatum* | Ericaceae | Shrub | 18.24 | ns | *atpB, matK, ndhF, rbcL, matR* | 138 fossils | ([Lu *et al.*, 2018](#_ENREF_18)) |
| *Lasianthus fordii* | Rubiaceae | Shrub | 18.37 | ns | *rps16, trnL-F,* ITS, ETS | One secondary calibration point and one fossil | ([Smedmark *et al.*, 2014](#_ENREF_26)) |
| *Elatostema stewardii* | Urticaceae | Herb | 19.24 | ns | *trnL-trnF, rpl14-rps8-infA-rpl36, matK, rbcL,* ITS, 18S, *matR* | Five fossils | ([Wu *et al.*, 2018](#_ENREF_35)) |
| *Magnolia delavayi* | Magnoliaceae | Tree | 19.51 | ns | *PHYA, LFY, GAI* | Three fossils | ([Nie *et al.*, 2008](#_ENREF_23)) |
| *Ardisia crenata* | Primulaceae | Shrub | 21.34 | ns | *atpB, matK, ndhF, rbcL, matR* | 138 fossils | ([Lu *et al.*, 2018](#_ENREF_18)) |
| *Ilex elmerrilliana* | Aquifoliaceae | Shrub | 21.92 | (16.59-30.1) | ITS, *nepGS* | Three fossils | ([Yao *et al.*, 2020](#_ENREF_39)) |
| *Dendropanax hainanensis* | Araliaceae | Tree | 26.87 | ns | *ndhF, trnL-F, rps16, atpB-rbcL, rpl16, psbA-trnH,* ITS | Two fossils | ([Li & Wen, 2013](#_ENREF_14)) |
| *crown age are shown; ns, not shown; Ma, million years ago; HPD, highest-probability-density interval. | | | | | | | |

| **Table S2** Divergence time estimation of 30 relict genera in East Asian subtropical evergreen broadleaved forests. Ev.=evergreen; Dec.=deciduous; Semi-Ev.=semi-evergreen; Sub-Ev.=sub-evergreen. | | | | | | | |
| --- | --- | --- | --- | --- | --- | --- | --- |
| Extant genus | Life form | Sampling | Marker | Crown age (Ma) | 95%HPD (Ma) | Calibrations | Reference |
| *Glyptostrobus* | Trees, Dec. | 1/1, *G. pensilis* | nuclear microsatellites | 0.33 | 0.16-0.49 | Substitution rate | ([Wu *et al.*, 2019](#_ENREF_34)) |
| *Ginkgo* | Trees, Dec. | 1/1, *G. biloba* | chloroplast genome | 0.39 | 0.22-0.79 | Eleveen fossils | ([Hohmann *et al.*, 2018](#_ENREF_6)) |
| *Piptanthus* | Shrubs, Ev. | 4/4, *P. concolor, P. laburnifolius, P. leiocarpus, P. tomentosus* | ITS | 0.83 | - | Seven fossils | ([Zhang, ML *et al.*, 2015](#_ENREF_45)) |
| *Pterostyrax* | Trees or shrubs, Dec. | 2/2, *P. corymbosus, P. psilophyllus* | *atpB, matK, ndhF, rbcL,matR* | 1.15 | - | 138 fossils | ([Lu *et al.*, 2018](#_ENREF_18)) |
| *Tapiscia* | Trees, Dec. | 2/2, *T. sinensis, T. yunnanensis* | *psbA-trnH, rpl32-trnL* | 1.33 | 1.02-1.74 | Substitution rate | ([Zhang, J *et al.*, 2015](#_ENREF_44)) |
| *Keteleeria* | Trees, Ev. | 8/~8, *K. xerophila, K. davidiana, K. evelyniana, K. fortunei, K. pubescens, K. hainanensis, K. calcarea, K. sp* | 27 nuclear genes | 1.9 | 0.4-3.4 | Substituution rate | ([Chou, 2020](#_ENREF_3)) |
| *Koelreuteria* | Trees, Dec. | 2/2, *K. paniculata, K. elegans* | *atpB, matK, ndhF, rbcL, matR* | 2.57 | - | 138 fossils | ([Lu *et al.*, 2018](#_ENREF_18)) |
| *Dipentodon* | Shrubs or trees, Semi-Ev. | 2/2, *D. longipedicellatus, D. sinicus* | *psbA-trnH, trnQ-rps16* | 2.97 | - | Substitution rate | ([Yuan *et al.*, 2008](#_ENREF_43)) |
| *Taiwania* | Trees, Ev. | 1/1, *T. cryptomerioides* | chloroplast DNA | 3.31 | 2.51-4.12 | Two fossils | ([Chou *et al.*, 2011](#_ENREF_4)) |
| *Diplopanax* | Trees, Ev. | 1/1, *D. stachyanthus* | *rps16, trnL-trnF, trnH-psbA* | 3.36 | 1.26-8.39 | Two fossils and one secondary calibration point | Estimated |
| *Pseudotaxus* | Shrubs, Ev. | 1/1, *P. chienii* | low-copy nuclear genes | 3.68 | 2.6-4.63 | Substitution rate | ([Kou *et al.*, 2020](#_ENREF_12)) |
| *Akebia* | Woody lianas, Dec. or Sub-Ev. | 3/4, *A. quinata, A. trifoliata, A. longeracemosa* | atpB, matK, ndhF, rbcL | 3.94 | 0.72-7.35 | Two secondary calibrations | Estimeted |
| *Davidia* | Trees, Dec. | 1/1, D. involucrata | *trnL–rpl32F, trnS–trnG* | 4.81 | 3.43-6.34 | Two fossils | ([Ma *et al.*, 2015](#_ENREF_19)) |
| *Emmenopterys* | Trees, Dec. | 1/1, *E. henryi* | *psbA–trnH, trnL–trnF, trnT–trnL* | 5.06 | 1.68-8.91 | One secondary calibration | ([Zhang, YH *et al.*, 2016](#_ENREF_48)) |
| *Cercidiphyllum* | Trees, Dec. | 2/2, *C. japonicum, C. magnificum* | *petA–psbJ, psbE–petL, rpl32–trnL, F71–R1516* | 5.32 | 1.93-9.25 | Two fossils | ([Qi *et al.*, 2012](#_ENREF_24)) |
| *Cunninghamia* | Trees, Ev. | 2/2, *C. lanceolata, C. konishii* | *rbcL, matK,* 18S, *PHYP* | 5.41 | 1.22-8.25 | Sixteen fossils | ([Leslie *et al.*, 2012](#_ENREF_13)) |
| *Euptelea* | Trees or shrubs, Dec. | 2/2, *E. pleiosperma, E. polyandra* | *rbcL, matK,* 26S | 5.46 | 1.23-10.87 | Four fossils | ([Cao *et al.*, 2016](#_ENREF_1)) |
| *Trochodendron* | Trees or shrubs, Ev. | 1/1, *T. aralioides* | *petG-trnP, petA-psbJ* | 5.5 | 2.7-8.3 | Substitution rate | ([Huang & Lin, 2006](#_ENREF_8)) |
| *Exbucklandia* | Trees, Ev. | 2/3, *E. tonkinensis, E. populnea* | *atpB, matK, ndhF, rbcL, matR* | 5.78 | - | 138 fossils | ([Lu *et al.*, 2018](#_ENREF_18)) |
| *Amentotaxus* | Shrubs or small trees, Ev. | 4/~6, *A. argotaenia, A. formosana, A. yunnanensis, A. poilanei* | *LFY, NFY, matk, rbcl* | 6.63 | 3.72-12.11 | One secondary calbrations and 5 fossils | ([Leslie *et al.*, 2012](#_ENREF_13)) |
| *Sargentodoxa* | Shrubs, climbing, Dec. | 1/1, *S. cuneata* | *matK* | 7.29 | 2.86-13.26 | Four fossils | ([Tian *et al.*, 2015](#_ENREF_30)) |
| *Pteroceltis* | Trees, Dec. | 1/1, *P. tatarinowii* | ITS, *trnL-trnF, trnH-psbA, rbcL* | 8.12 | 4.17-12.99 | Two fossils | Estimated |
| *Tetracentron* | Trees, Dec. | 1/1, *T. sinense* | *petG–trnP, psbK–psbI, rps2, rpl16, psbA–trnH, rpl32–trnL, petA–psbJ, petL–psbE* | 9.6 | 2.2-27.0 | One secondary calibration | ([Sun, YX *et al.*, 2014](#_ENREF_29)) |
| *Dipelta* | Shrubs, Dec. | 3/3, *D. yunnanensis, D. floribunda, D. elegans* | ITS, *matK, rbcL, trnL-F, trnS-G, ndhA, petB-D, psbA-trnH, psbM-trnD, trnL-rpl32* | 9.92 | 1.83-20.85 | Three fossils | ([Wang *et al.*, 2015](#_ENREF_32)) |
| *Ostryopsis* | Shrubs, Dec. | 3/3, *O. intermedia, O. davidiana, O. nobilis* | ITS, *matK and rbcL,* 11 nuclear gene sequences and 20 nuclear microsatellites | 9.95 | 6-11.24 | Two fossils/substitution rate | ([Liu *et al.*, 2014](#_ENREF_16)) |
| *Dipteronia* | Trees, Dec. | 2/2, *D. dyerana, D. sinensis* | *atpB, matK, ndhF, rbcL,matR* | 10.97 | - | 138 fossils | ([Lu *et al.*, 2018](#_ENREF_18)) |
| *Semiliquidambar* | Trees, Dec. or Ev. | 2/3, *S. cathayensis, S. chingii* | *atpB, matK, ndhF, rbcL,matR* | 13.74 | - | 138 fossils | ([Lu *et al.*, 2018](#_ENREF_18)) |
| *Cyclocarya* | Trees, Dec. | 1/1, *C. paliurus* | *atpB–rbcL, psbA–trnH* | 16.69 | 8.42-27.86 | Four fossils | ([Kou *et al.*, 2016](#_ENREF_11)) |
| *Cephalotaxus* | Trees or shrubs, Ev. | 11/~13, *C. lanceolata, C. fortunei, C. sinensis, C. latifolia, C. griffithii, C. hainanensis, C. mannii, C. wilsoniana, C. harringtonia, C. oliveri, C. koreana* | *rbcL, matK,* 18S, *PHYP* | 19.48 | 12.18-27.74 | Sixteen fossils | ([Leslie *et al.*, 2012](#_ENREF_13)) |
| *Hovenia* | Trees or shrubs, Dec. | 3/3, *H. trichocarpa, H. dulcis, H. acerba*) | ITS, *trnL-F, rbcL* | 35.91 | 24.23-49.31 | One fossil | ([Chen *et al.*, 2017](#_ENREF_2)) |

Ma, million years ago; HPD, highest-probability-density interval.

**
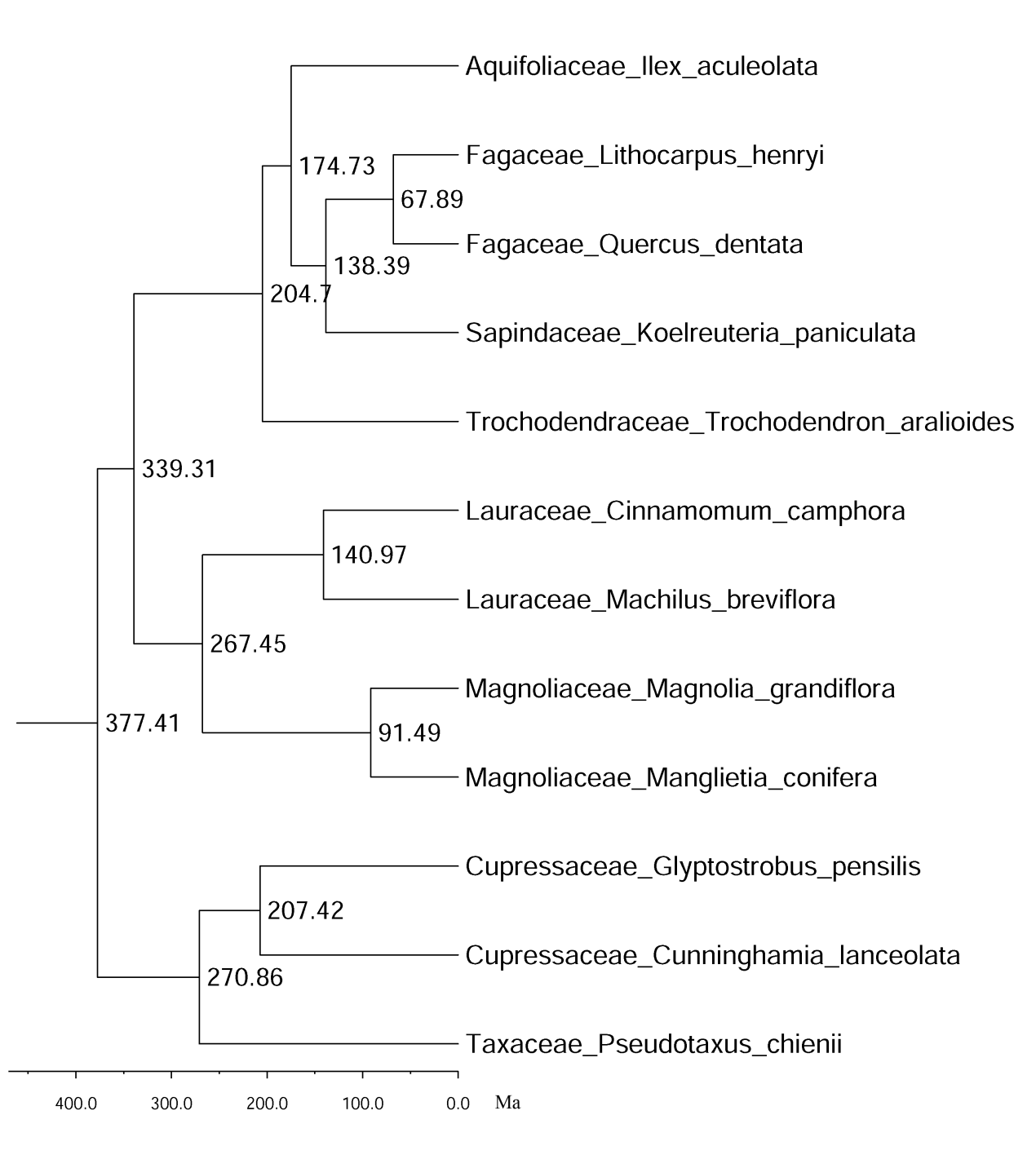
**

**Figure S1** Calibrated-phylogeny of some characteristic taxa in East Asian evergreen broadleaved forests.

**Reference**

**Cao YN, Comes HP, Sakaguchi S, Chen LY, Qiu YX. 2016.** Evolution of East Asia’s Arcto-Tertiary relict *Euptelea* (Eupteleaceae) shaped by Late Neogene vicariance and Quaternary climate change. *BMC Evolutionary Biology* **16**: 1-17.

**Chen YS, Meseguer AS, Godefroid M, Zhou Z, Zhang JW, Deng T, Kim JH, Nie ZL, Liu YS, Sun H. 2017.** Out-of-India dispersal of *Paliurus* (Rhamnaceae) indicated by combined molecular phylogenetic and fossil evidence. *Taxon* **66**: 1-13.

**Chou SY. 2020.** Genetic divergence between *Keteleeria* species (Pinaceae) using multilocus analysis. *Unpublished*.

**Chou YW, Thomas PI, Ge XJ, LePage BA, Wang CN. 2011.** Refugia and phylogeography of Taiwania in East Asia. *Journal of Biogeography* **38**: 1992-2005.

**Deng M, Jiang XL, Hipp AL, Manos PS, Hahn M. 2017.** Phylogeny and biogeography of East Asian evergreen oaks (*Quercus* section *Cyclobalanopsis* ; Fagaceae): Insights into the Cenozoic history of evergreen broad-leaved forests in subtropical Asia. *Molecular Phylogenetics and Evolution* **119**: 170-181.

**Hohmann N, Wolf EM, Rigault P, Zhou W, Kiefer M, Zhao Y, Fu CX, Koch MA. 2018.** *Ginkgo biloba*’s footprint of dynamic Pleistocene history dates back only 390,000 years ago. *BMC Genomics* **19**: 299.

**Huang JF, Li L, van der Werff H, Li HW, Rohwer JG, Crayn DM, Meng HH, van der Merwe M, Conran JG, Li J. 2016.** Origins and evolution of cinnamon and camphor: A phylogenetic and historical biogeographical analysis of the *Cinnamomum* group (Lauraceae). *Molecular Phylogenetics and Evolution* **96**: 33-44.

**Huang SF, Lin TP. 2006.** Migration of *Trochodendron aralioides* (Trochodendraceae) in Taiwan and Its Adjacent Areas. *Botanical Studies* **47**: 83-88.

**Janssens SB, Groeninckx I, De Block PJ, Verstraete B, Smets EF, Dessein S. 2016.** Dispersing towards Madagascar: Biogeography and evolution of the Madagascan endemics of the Spermacoceae tribe (Rubiaceae). *Molecular Phylogenetics and Evolution* **95**: 58-66.

**Kim C, Cameron KM, Kim JH. 2017.** Molecular systematics and historical biogeography of *Maianthemum* s.s. *American Journal of Botany* **104**: 939-952.

**Kou YX, Cheng SM, Tian S, Li B, Fan DM, Chen YJ, Soltis DE, Soltis PS, Zhang ZY. 2016.** The antiquity of *Cyclocarya paliurus* (Juglandaceae) provides new insights into the evolution of relict plants in subtropical China since the late Early Miocene. *Journal of Biogeography* **43**: 351-360.

**Kou YX, Zhang L, Fan DM, Cheng SM, Li DZ, Hodel RGJ, Zhang ZY. 2020.** Evolutionary history of a relict conifer, *Pseudotaxus chienii* (Taxaceae), in southeast China during the late Neogene: old lineage, young populations. *Annals of Botany* 125: 105-117.

**Leslie AB, Beaulieu JM, Rai HS, Crane PR, Donoghue MJ, Mathews S. 2012.** Hemisphere-scale differences in conifer evolutionary dynamics. *Proceedings of the National Academy of Sciences of the USA* **109**: 16217-16221.

**Li R, Wen J. 2013.** Phylogeny and Biogeography of *Dendropanax* (Araliaceae), an Amphi-Pacific Disjunct Genus between Tropical/Subtropical Asia and the Neotropics. *Systematic Botany* **38**: 536-551.

**Li Y, Yan HF, Ge XJ. 2012.** Phylogeographic analysis and environmental niche modeling of widespread shrub *Rhododendron simsii* in China reveals multiple glacial refugia during the last glacial maximum. *Journal of Systematics and Evolution* **50**: 362-373.

**Liu BB, Abbott RJ, Lu ZQ, Tian B, Liu JQ. 2014.** Diploid hybrid origin of *Ostryopsis intermedia* (Betulaceae) in the Qinghai-Tibet Plateau triggered by Quaternary climate change. *Molecular Ecology* **23**(12): 3013-3027.

**Liu XL, Wen J, Nie ZL, Johnson G, Liang ZS, Chang ZY. 2013.** Polyphyly of the *Padus* group of *Prunus* (Rosaceae) and the evolution of biogeographic disjunctions between eastern Asia and eastern North America. *Journal of Plant Research* **126**: 351-361.

**Lu LM, Mao LF, Yang T, Ye JF, Liu B, Li HL, Sun M, Miller JT, Mathews S, Hu HH, Niu YT, Peng DX, Chen YH, Smith SA, Min C, Xiang KL, Le CT, Dang VC, Lu AM, Soltis PS, Soltis DE, Li JH, Chen ZD. 2018.** Evolutionary history of the angiosperm flora of China. *Nature* **554**: 234-238.

**Ma Q, Du Y, Chen N, Zhang L, Li J, Fu CX. 2015.** Phylogeography of *Davidia involucrata* (Davidiaceae) Inferred from cpDNA Haplotypes and nSSR Data. *Systematic Botany* **40**: 769-810.

**Martín-Bravo S, Jiménez-Mejías P, Villaverde T, Escudero M, Hahn M, Spalink D, Roalson EH, Hipp AL, Benítez-Benítez C, Bruederle LP, Fitzek E, Ford BA, Ford KA, Garner M, Gebauer S, Hoffmann MH, Jin XF, Larridon I, Léveillé-Bourret É, Lu Y-F, Luceño M, Maguilla E, Márquez-Corro JI, Míguez M, Naczi R, Reznicek AA, Starr JR. 2019.** A tale of worldwide success: Behind the scenes of *Carex* (Cyperaceae) biogeography and diversification. *Journal of Systematics and Evolution* **57**: 695-718.

**Morris AB, Bell CD, Clayton JW, Judd WS, Soltis DE, Soltis PS. 2007.** Phylogeny and divergence time estimation in *Ilicium* with implications for new world biogeography. *Systematic Botany* **32**: 236-249.

**Nie ZL, Sun H, Manchester SR, Meng Y, Luke Q, Wen J. 2012.** Evolution of the intercontinental disjunctions in six continents in the *Ampelopsis* clade of the grape family (Vitaceae). *BMC Evolutionary Biology* **12**: 17.

**Nie ZL, Wen J, Azuma H, Qiu YL, Sun H, Meng Y, Sun WB, Zimmer EA. 2008.** Phylogenetic and biogeographic complexity of Magnoliaceae in the Northern Hemisphere inferred from three nuclear data sets. *Molecular Phylogenetics and Evolution* **48**: 1027-1040.

**Qi XS, Chen C, Comes HP, Sakaguchi S, Liu YH, Tanaka N, Sakio H, Qiu YX. 2012.** Molecular data and ecological niche modelling reveal a highly dynamic evolutionary history of the East Asian Tertiary relict *Cercidiphyllum* (Cercidiphyllaceae). *New Phytologist* **196**: 617-630.

**Sakaguchi S, Qiu YX, Liu YH, Qi XS, KIM SH, Takeuchi Y, Worth JR, Yamasaki M, Sakurai S. 2012.** Climate oscillation during the Quaternary associated with landscape heterogeneity promoted allopatric lineage divergence of a temperate tree *Kalopanax septemlobus* (Araliaceae) in East Asia. *Molecular Ecology* **21**: 3823-3838.

**Smedmark JEE, Razafimandimbison SG, Wikström N, Bremer B. 2014.** Inferring geographic range evolution of a pantropical tribe in the coffee family (Lasiantheae, Rubiaceae) in the face of topological uncertainty. *Molecular Phylogenetics and Evolution* **70**: 182-194.

**Su YJ, Liao WB, Wang T, Sun YF, Wei Q, Chang HT. 2011.** Phylogeny and evolutionary divergence times in *Apterosperma* and *Euryodendron*: Evidence of a Tertiary origin in south China. *Biochemical Systematics and Ecology* **39**: 769-777.

**Sun Y, Hu HQ, Huang HW, Vargas-Mendoza CF. 2014.** Chloroplast diversity and population differentiation of *Castanopsis fargesii* (Fagaceae): A dominant tree species in evergreen broad-leaved forest of subtropical China. *Tree Genetics and Genomes* **10**: 1531-1539.

**Sun YX, Moore MJ, Yue LL, Feng T, Chu HJ, Chen ST, Ji YH, Wang HC, Li JQ. 2014.** Chloroplast phylogeography of the East Asian Arcto-Tertiary relict *Tetracentron sinense* (Trochodendraceae). *Journal of Biogeography* **41**: 1721-1732.

**Tian S, Lei SQ, Wan H, Deng LL, Bo L, Meng QL, Soltis DE, Soltis PS, Fan DM, Zhang ZY. 2015.** Repeated range expansions and inter-/postglacial recolonization routes of *Sargentodoxa cuneata* (Oliv.) Rehd. et Wils. (Lardizabalaceae) in subtropical China revealed by chloroplast phylogeography. *Molecular Phylogenetics and Evolution* **85**: 238-246.

**Wang GY, Yang YP. 2018.** Hypothesizing the origin, migration routes, and distribution patterns of *Ophiopogon* (Asparagaceae) in East and Southeast Asia. *Journal of Systematics and Evolution* **56**: 194-201.

**Wang HF, Landrein S, Dong WP, Nie ZL, Kondo K, Funamoto T, Wen J, Zhou SL, Lumbsch HT. 2015.** Molecular Phylogeny and Biogeographic Diversification of Linnaeoideae (Caprifoliaceae s. l.) Disjunctly Distributed in Eurasia, North America and Mexico. *PLoS ONE* **10**: e0116485.

**Weeks A, Zapata F, Pell SK, Daly DC, Mitchell JD, Fine PVA. 2014.** To move or to evolve: contrasting patterns of intercontinental connectivity and climatic niche evolution in "Terebinthaceae" (Anacardiaceae and Burseraceae). *Frontiers in Genetics* **5**: 409.

**Wu XT, Ruhsam M, Wen Y, Thomas PI, Worth JRP, Lin XY, Wang MQ, Li XY, Chen L, Lamxay V, Le Canh N, Coffman G. 2019.** The last primary forests of the Tertiary relict *Glyptostrobus pensilis* contain the highest genetic diversity. *Forestry* **93**: 359-375.

**Wu ZY, Liu J, Provan J, Wang H, Chen CJ, Cadotte MW, Luo YH, Amorim BS, Li DZ, Milne RI. 2018.** Testing Darwin's transoceanic dispersal hypothesis for the inland nettle family (Urticaceae). *Ecology Letters* **21**: 1515-1529.

**Xie L, Yi TS, Li R, Li DZ, Wen J. 2010.** Evolution and biogeographic diversification of the witch-hazel genus (*Hamamelis* L., Hamamelidaceae) in the Northern Hemisphere. *Molecular Phylogenetics and Evolution* **56**: 675-689.

**Xu J, Deng M, Jiang XL, Westwood M, Song YG, Turkington R. 2015.** Phylogeography of *Quercus glauca* (Fagaceae), a dominant tree of East Asian subtropical evergreen forests, based on three chloroplast DNA interspace sequences. *Tree Genetics and Genomes* **1**: 1-17.

**Yang CK, Chiang YC, Huang BH, Ju LP, Liao PC. 2018.** Nuclear and chloroplast DNA phylogeography suggests an Early Miocene southward expansion of *Lithocarpus* (Fagaceae) on the Asian continent and islands. *Botanical Studies* **59**: 27.

**Yao X, Song Y, Yang JB, Tan YH, Corlett RT. 2020.** Phylogeny and biogeography of the hollies (*Ilex* L., Aquifoliaceae). *Journal of Systematics and Evolution* **Online**.

**Ye JW, Li DZ, Hampe A. 2019.** Differential Quaternary dynamics of evergreen broadleaved forests in subtropical China revealed by phylogeography of *Lindera aggregata* (Lauraceae). *Journal of Biogeography* **46**: 1112-1123.

**Yu CC, Chung KF. 2017.** Why *Mahonia*? Molecular recircumscription of *Berberis* s.l., with the description of two new genera, *Alloberberis* and *Moranothamnus*. *Taxon* **66**: 1371-1392.

**Yu XQ, Gao LM, Soltis DE, Soltis PS, Yang JB, Fang L, Yang SX, Li DZ. 2017.** Insights into the historical assembly of East Asian subtropical evergreen broadleaved forests revealed by the temporal history of the tea family. *New Phytologist* **215**: 1235-1248.

**Yuan QJ, Zhang ZY, Peng H, Ge S. 2008.** Chloroplast phylogeography of *Dipentodon* (Dipentodontaceae) in southwest China and northern Vietnam. *Molecular Ecology* **17**: 1054-1065.

**Zhang J, Li Z, Fritsch PW, Tian H, Yang A, Yao X. 2015.** Phylogeography and genetic structure of a Tertiary relict tree species, *Tapiscia sinensis* (Tapisciaceae): Implications for conservation. *Annals of Botany* **116**: 1-11.

**Zhang ML, Huang JF, Sanderson SC, Yan P, Wu YH, Pan BR. 2015.** Molecular Biogeography of Tribe Thermopsideae (Leguminosae): A Madrean-Tethyan Disjunction Pattern with an African Origin of Core Genistoides. *Biomed Research International* **2015**: 864804.

**Zhang Q, Feild TS, Antonelli A. 2015.** Assessing the impact of phylogenetic incongruence on taxonomy, floral evolution, biogeographical history, and phylogenetic diversity. *American Journal of Botany* **102**: 566-580.

**Zhang XZ, Zeng CX, Ma PF, Haevermans T, Zhang YX, Zhang LN, Guo ZH, Li DZ. 2016.** Multi-locus plastid phylogenetic biogeography supports the Asian hypothesis of the temperate woody bamboos (Poaceae: Bambusoideae). *Molecular Phylogenetics and Evolution* **96**: 118-129.

**Zhang YH, Wang IJ, Comes HP, Hua P, Qiu YX. 2016.** Contributions of historical and contemporary geographic and environmental factors to phylogeographic structure in a Tertiary relict species, *Emmenopterys henryi* (Rubiaceae). *Scientfic Reports* **6**: 24041.
